# Clinical taxonomy of Subjective Cognitive Decline: evidence from a Memory Clinic Cohort

**DOI:** 10.1002/alz70857_098967

**Published:** 2025-12-24

**Authors:** Federica Ribaldi, Christian Chicherio, Augusto J. Mendes, Giovanni Frisoni

**Affiliations:** ^1^ Geneva Memory Center, Department of Rehabilitation and Geriatrics, Geneva University Hospitals, Geneva, Geneva, Switzerland; ^2^ Geneva University Hospitals, Geneva, Geneva, Switzerland; ^3^ Laboratory of Neuroimaging of Aging (LANVIE), University of Geneva, Geneva, Switzerland; ^4^ University Hospitals and University of Geneva, Geneva, Switzerland

## Abstract

**Background:**

Subjective cognitive decline (SCD) is recognized as a potential early risk factor for Alzheimer's disease (AD) and other dementias, but standardized approaches to assess and address the concerns of individuals with SCD remain limited. Moreover, its prevalence is increasing in memory clinics, highlighting an unmet clinical need for tailored protocols and evidence‐based guidance. A recent work proposed a taxonomy for SCD, categorizing individuals into three subgroups based on psychological factors (e.g., anxiety, depression, neuroticism), comorbidities (e.g., vascular risk factors, neurological or somatic comorbidities), and no apparent cause. This taxonomy provides a structured framework for interpreting SCD and guiding management strategies. However, its clinical applicability requires validation in independent cohorts.

**Method:**

The SCD taxonomy was initially tested on a small subset of individuals from the Geneva Memory Center, classifying individuals into the three previously introduced subgroups using detailed clinical reports (Ribaldi et al., 2024). Currently, the taxonomy is being applied to a larger retrospective cohort. All participants underwent comprehensive neuropsychological assessments and clinical evaluations, with a subset also featuring biomarker data. To streamline its application, the taxonomy has been converted into an automated algorithm that assigns individuals to subgroups based on available clinical data. The ongoing effort focuses on validating the accuracy of this automated classification by comparing it with expert clinical judgments. Once this step is complete, baseline differences among the subgroups will be analyzed, followed by an assessment of longitudinal cognitive trajectories.

**Result:**

We will present the taxonomy structure and preliminary findings from the validation cohort. Initial findings from the original cohort demonstrated the taxonomy's ability to stratify individuals based on psychological factors and somatic comorbidities, revealing significant differences in demographics, clinical features, and cognitive trajectories across subgroups. Specifically, the SCD group with no apparent cause exhibited faster cognitive decline and an AD‐like biomarker profile. Ongoing analyses aim to confirm these findings in the larger cohort.

**Conclusion:**

Preliminary findings support the applicability of the SCD taxonomy, underscoring its potential as a clinical tool for risk stratification and personalized intervention. This validation represents a critical step toward integrating the taxonomy into routine clinical workflows, addressing the growing needs of individuals with SCD.

